# Essential Mineral Elements and Potentially Toxic Elements in Orange-Fleshed Sweet Potato Cultivated in Northern Ethiopia

**DOI:** 10.3390/biology12020266

**Published:** 2023-02-07

**Authors:** Gloria Peace Lamaro, Yemane Tsehaye, Atkilt Girma, Andrea Vannini, Riccardo Fedeli, Stefano Loppi

**Affiliations:** 1 Institute of Climate and Society, Mekelle University, Mekelle P.O. Box 231, Ethiopia; 2 Department of Dryland Crops and Horticultural Sciences, College of Dryland Agriculture and Natural Resources, Mekelle University, Mekelle P.O. Box 231, Ethiopia; 3 Department of Land Resources Management and Environmental Protection (LaRMEP), College of Dryland Agriculture and Natural Resources, Mekelle University, Mekelle P.O. Box 231, Ethiopia; 4 Department of Life Sciences, University of Siena, 53100 Siena, Italy; 5 BAT Center—Interuniversity Center for Studies on Bioinspired Agro-Environmental Technology, University of Naples Federico II, 80138 Napoli, Italy

**Keywords:** recommended dietary allowance, health risks, essential mineral elements, orange-fleshed sweetpotato, potentially toxic elements

## Abstract

**Simple Summary:**

This study aimed to investigate the influence of the agro-climatic environment of Northern Ethiopia on the content of essential mineral elements of selected orange-fleshed sweetpotato genotypes, the potential contribution of each genotype’s essential mineral elements to the required daily allowance, and the potential risk to human health from the accumulation of potentially toxic elements in the tuberous roots. The results showed consistent interspecific variability as well as important and significant intraspecific differences, which could depend on genotype and environment. Four of the five investigated genotypes can provide an amount of several essential mineral elements high enough to meet 100% of the recommended dietary allowance for all age groups ≤8 years. For all the genotypes, no health risks associated with their consumption emerged for Cr, As, and Pb, but Al, Cd, Cu, Fe, Mn and Ni is > 1 showed evidence of greater health risk, especially in children.

**Abstract:**

This study investigated the influence of the agro-climatic environment of Northern Ethiopia on the content of essential mineral elements of selected orange-fleshed sweetpotato genotypes, the potential contribution of each genotype’s essential mineral elements to the recommended dietary allowance, and the potential risk to human health from the accumulation of potentially toxic elements in the tuberous roots of the studied genotypes. The results showed consistent interspecific variations in the content of essential mineral elements among the studied orange-fleshed sweetpotato genotypes, as well as important intraspecific differences, which could depend on the variations in soil mineral and organic matter content, rainfall, temperature, as well as interactions between genotype and environment. The investigated genotypes, especially Kulfo, Ininda, Gloria, and Amelia, can provide an amount of several essential mineral elements high enough to meet 100% of the recommended dietary allowance for all age groups ≤8 years. The mean content of potentially toxic elements in tuberous roots and their daily intake values were within the recommended permissible levels; likewise, no health risk was associated with the consumption of these genotypes for Cr, As, and Pb. However, Al, Cd, Cu, Fe, Mn, and Ni is > 1, consumption imposes health risks based on daily accumulation.

## 1. Introduction

Sweetpotato (*Ipomoea batatas* (L.) Lam.) is globally ranked as the sixth major food crop, and it is the fifth most important food crop in Sub-Saharan Africa [1]. The tuberous roots of this crop plant are rich in essential mineral elements such as Ca, Cu, Fe, K, Mn, P, and Zn [2,3], which are necessary for humans’ metabolic and biological functions. Mineral element deficiency can result in many disorders [4], and is associated with high mortality, high disease occurrence, and infertility [5]. 

The concentration of essential minerals elements in crops can be increased through biofortification [6,7,8]. When biofortifying sweetpotato, special care should be given to both the nutrient content and genotype-by-environment interactions (GEIs) of these essential mineral elements [7]. Strong GEIs in orange-fleshed sweetpotato have been reported by several authors [9,10,11]. According to Laurie et al. [9], variations in sweetpotato mineral content could be due to differences in GEIs, soil mineral content, and pH. Similarly, the content of essential mineral elements in sweetpotato may vary due to soil contamination with potentially toxic elements (PTEs) [12,13], as a result of anthropogenic activities such as mining, fossil fuel combustion, industrial activities, or heavy use of biosolids in agriculture [14]. Other major sources of PTEs are road transportation [15] and gasoline stations [16].

Several PTEs, e.g., Al, As, Cd, Pb, and Hg, have no known biological role and are thus harmful to humans [17], while other PTEs such as Cu, Cr, Fe, Ni, and Zn are important factors of metabolic functioning but become toxic at high concentrations [18,19]. In common crop plants such as onion, broad bean, tobacco, and potato, PTE toxicity induces inhibition of photosynthesis and growth, low cell division, and impairment of nucleic acid [20,21,22,23] and interferes with uptake and translocation of nitrogen [24], as well as other essential minerals [25,26]. 

Crop plants grown in heavily contaminated soils can potentially accumulate high concentrations of PTEs, which can thus enter the food chain, with negative implications for human and animal bodies [19,27]. Accumulation of toxic elements in humans may induce damage to several organs, even at low levels of exposure [28]. They are associated with cancer, DNA damage through mutation, deletion, or oxygen radical attack on DNA structure [29]. 

In African countries such as Ethiopia, war and other anthropogenic activities may cause severe soil pollution of agricultural soils. There is therefore a need to evaluate the qualities of agricultural commodities, including sweetpotatoes, in terms of both essential minerals and PTEs for food and nutritional security, given their great importance as a source of food for the local population.

This study thus aimed to investigate: (I) the influence of the agro-climatic environment on the content of essential mineral elements of selected orange-fleshed sweetpotato (OFSP) genotypes, (II) the potential contribution of each genotype’s essential mineral elements to the required daily allowance, and (III) the potential risk to human health from the accumulation of PTEs in the tuberous roots of the studied genotypes.

## 2. Materials and Methods

### 2.1. Study Sites

The experiments were run at three sites: Mekelle-1 (M1), Mekelle-2 (M2), and Aba’ala (A). The main climatic, geographical, and soil characteristics of the study sites are shown in Table 1. Africa has been divided into agro-climatic zones based on of the length of the growing season, which is associated with rainfall [30]; M1 and M2 fall within the semi-arid agro-climatic zone, while A falls within the arid agro-climatic zone. The arid agro-climatic zone has a growing period shorter than 60 days, while the semi-arid agro-climatic zone is 60–120 days [30]. At each site, experimental plots of 11 × 17 m (approximately 187 m^2^) were randomly selected.

### 2.2. Planting Material

A total of 5 distinct OFSP genotypes: 1 = Amelia, 2 = Gloria, 3 = Ininda, 4 = Kulfo, and 5 = Melinda, were obtained from the Tigray Agricultural Research Institute (TARI). These planting materials (except Kulfo (release-check)) were from the new germplasm sourced from Mozambique for trials at TARI in Ethiopia.

Planting was carried out at the three experimental sites using a randomized complete block design with three replicates at a spacing of 0.3 m × 1.0 m. The space between replication blocks was 1.0 m, and each plot within a replicate was 0.5 m. Each replication block contained five plots, and each plot was planted with 30 stems of a genotype.

The experiment depended on the natural environmental conditions for moisture and minerals. Nevertheless, at Aba’ala (arid agro-climate) water was supplied for two weeks at initial planting to enable crop pickup due to a lack of rainfall. No supplementary irrigation was provided at the other two sites. Weed control was achieved through mechanical management until the establishment of a full canopy cover of the crop.

### 2.3. Tuberous Roots

At maturation (at the end of November 2020), ten plants were randomly selected from the middle of the experimental plots to avoid border influence, and their tuberous roots were harvested. One tuberous root from each plant from the harvestable plot of each genotype was randomly taken to make a composite sample of each genotype used for the nutrient element quality and the content of PTEs analysis. Samples were washed in deionized water, peeled, sliced, and then freeze-dried (True-Ten Industrial Co., Taichung City, Taiwan), milled (Mini Mill, Thomas Scientific, Swedesboro, NJ, USA), and stored at −24 °C until chemical analysis.

#### Chemical Analysis

About 200 mg of ground samples for each OFSP genotype were mineralized with a mixture of 3 cm^3^ of 70% (*v*/*v*) HNO_3_, 0.2 cm^3^ of 50% (*v*/*v*) HF, and 0.5 cm^3^ of 30% (*v*/*v*) H_2_O_2_, using the microwave digestion system Ethos 900 (Milestone, Sorisoles, Italy) at 280 °C and 5.5 MPa, following the method described by Loppi et al. [31]. The content of Al, Ca, Cd, Cr, Cu, Fe, K, Mg, Mn, Ni, P, Pb, and Zn was quantified by an ICP-MS NexION 350 (Perkin Elmer, Waltham, MA, USA). The analytical quality was measured using the certified standard reference material DC 73350 “Poplar leaves” (Analytika, Praque, Czech Republic); recoveries were 94–112%. The precision of analyses was estimated by the coefficient of variation of 5 replicates and was always >97%. The results were expressed on a dry weight basis.

### 2.4. Data Analysis

The mineral contribution of each genotype to the recommended dietary allowance (RDA) or to the recommended adequate intake (RAI) for humans for Ca, Cu, Fe, K, Mg, Mn, Na, P, and Zn was calculated using a daily intake of 250 g of dry weight (dw) OFSP tuberous roots in mg per day, making it directly comparable to the standard consumption estimates. The average weight, height, and body mass for different age groups used to determine the RDA/RAI for different essential nutrients is taken from U.S. National Academy of Sciences [32] (Table 2).

The average essential minerals element content per genotype from the three environments was used to calculate the genotypes’ RDA contribution. The reference RDA/RAI are 270–1300 mg Ca, 0.4–1.3 mg Cu, 40–45 mg Fe, 400–1300 mg K, 75–360 mg Mg, and 0.6–11 mg Mn, 400–1300 mg Na, 275–1250 mg P, and 3–14 mg Zn [32,33]. For each element, the corresponding percentage contribution to the RDA/RAI was calculated as the mineral content in 250 g (dw)/RDA of each age group ×100.

To evaluate the health risk from PTEs associated with the consumption of OFSP, the Health Risk Index (*HRI*) was calculated according to the formula:

(1) $$HRI=\frac{DIPTE}{Rfd}$$

The value of *DIPTE* (Daily Intake of PTE) was calculated using the formula proposed by Khan et al. [34]:

(2) $$DIPTE=\frac{COFSP\times Cfactor\;\times \;Dfood\;intake}{Baverage\;weight}$$

where: C_OFSP_ is the average concentration (mg kg^−1^) of PTE in OFSP samples; D_food intake_ is the daily consumption of OFSP (250 g); B_average_ weight is the average body weight (70 kg). C_factor_ is the conversion factor 0.085.

The values of R_fd_ (reference dose in mg kg^−1^ day^−1^) for the investigated PTEs were Al = 1.0, As = 0.07, Cd = 0.001, Cr = 1.5, Cu = 0.04, Fe = 0.7, Mn = 0.014, Ni = 0.02, Pb = 0.025, and Zn = 0.3. These R_fd_ values were taken from the IRIS (Integrated Risk Information System) of the US EPA [35,36]. A value of *HRI* > 1 for a given PTE is indicative of a potential risk to human health [37].

The data approached a normal distribution (Shapiro–Wilk test, *p* < 0.05), and were explored for significant differences (*p* < 0.05) across sweetpotato genotype and environment using two-way ANOVA followed by Tukey’s test for post hoc comparisons. All calculations were run with the free software R [38].

## 3. Results

### 3.1. Mineral Elements

The mean values of essential mineral elements are reported in Table 3. Sweet potatoes from Aba’ala had by far the highest Ca, K, and P content; Cu, Ni, and Mn were also higher at Aba’ala. Trends in genotypes were element specific.

The results of two-way ANOVA (Table 4) showed that variations in genotype, environment, and the interactions between them are almost always statistically significant (*p* < 0.05), with exceptions such as Ni and Zn for genotype; Cr, Mg, and Zn for environment; and Cr, Ni, P, and Zn for the interactions.

The recommended dietary allowance (RDA)/recommended adequate intake (RAI) contributions for the different age groups are generally high for all mineral elements, with exceptions being Mg, Ca, Cr, Fe, Mn, Na, P, and Zn (Table 5).

### 3.2. Potentially Toxic Elements (PTEs)

The mean PTEs values are reported in Table 6. Melinda has the highest content of Cu and Fe and Amelia for Mn. Gloria ranked first for Al, Cr. Ininda had a high mean Cu content, and Kulfo had a low mean content of Al, Fe, and Mn. Environmental differences did not emerge for Al, Cd, Cr, Pb, and Zn. Aba’ala had a high content of Cu, Mn, and Ni, while Mekelle-1 and Makelle-2 had high contents of As and Fe, respectively. Tukey’s test (95%) significant confidence showed variations in the mineral element content in genotypes for almost all elements except Cd, Ni, Pb, and Ni; meanwhile, in terms of environment, the concentration of mineral elements varied, except for Al, Cd, Cr, Pb, and Zn. 

The results of two-way ANOVA (Table 7) showed that genotype was only statistically significant (*p* < 0.05) for Al and Cr, Cu, Fe, and Mn, while the environment was significant only for As, Cu, Fe, Mn, and Ni. Only Cu, Fe, and Mn showed significant genotype x environment interactions.

### 3.3. Daily Intake of Potential Toxic Elements (DIPTEs) and Health Risk Index (HRI)

On average, the values of the DIPTEs followed the order Fe > Al > Zn > Cu > Mn > Ni > Cd > Cr > Pb > As in the five genotypes (Table 8). In some cases (Amelia, Melinda) the order of Cu and Mn was reversed. 

The values of the *HRI* (Table 9) showed that Mn, Ni, Cu, Pb, Zn, and Cd were higher than the rest of the studied PTEs. On average, the order of the *HRI* values was Mn > Cd > Cu > Ni > Fe > Zn > Al > Pb > As > Cr.

## 4. Discussion

In Ethiopia, sweetpotato breeding programs prioritize improving in beta-carotene, yield, and resistance to pests and diseases. In this study, we evaluated the response of five genotypes to their environments in terms of concentrations of essential mineral and PTEs, with the aim of boosting nutritional security in Northern Ethiopia.

This study showed a wide array of variation in the content of mineral elements related to the effect of both genotype and environment, and the interactions between them. The overall high mean mineral content in Aba’ala (arid lowland) agrees with other studies, e.g., Laurie et al. [9] that reported high mineral content in sweetpotato varieties from lowland compared to their counterparts at highland. 

All genotypes could provide more than 100% RDA/RAI for age groups ≤12 months and at least more than 50% RDA/RAI for age groups ≥8 years. All genotypes provided 100% RDA/RAI for all the different age groups for Cu and K with 250 g of dry weight, indicating that all the studied genotypes have very high Cu and K content. Generally, Ca, Cr, Mn, Fe, Na, Zn, and P content was low: all genotypes were unable to contribute >50% RDA/RAI for age groups ≥8 years. A similar low Mn content was already reported by Tumwegamire [10] and Tumwegamire et al. [39].

The concentration range for most elements in the studied OFSP tuberous roots (Al = 16.6–21.8 mg kg^−1^, As = 0.025–0.033 mg kg^−1^, Cr = 0.15–0.24 mg kg^−1^, Cu = 5.9–7.7 mg kg^−1^, Fe = 40.0–43.8 mg kg^−1^, Mn = 4.7–8.5 mg kg^−1^, Pb = 0.40–0.63 mg kg^−1^, Zn = 11.4–16.3 mg kg^−1^) was lower than the concentration reported for the “reference plant” [40] (Al = 80 mg kg^−1^, As = 0.1 mg kg^−1^, Cr = 1.5 mg kg^−1^, Cu = 10 mg kg^−1^, Fe = 150 mg kg^−1^, Mn = 200 mg kg^−1^, Pb = 1 mg kg^−1^, Zn = 50 mg kg^−1^). Nevertheless, the concentration ranges of Cd (0.12–0.22 mg kg^−1^) and Ni (1.8–2.7 mg kg^−1^) were slightly higher than those in the “reference plant” (Cd = 0.05 mg kg^−1^, Ni = 1.5 mg kg^−1^). However, this concentration may pose negligible health risks on a daily intake basis [40,41,42,43,44,45]. 

The calculated DIPTEs for all elements were within the estimated permissible daily intake levels [36,44]. Similarly, values of the health risk index of the studied potentially toxic elements through the intake of orange-fleshed sweetpotato (dry weight) from different studied genotypes were within the safe limit < 1 for As, Cr, and Pb, but Al, Cd, Cu, Fe, Mn, and Ni [34,37]. Thus, it can be suggested that all the studied orange-fleshed sweetpotato genotypes can be safely consumed as far as the health risk from exposures to PTEs As, Cr, and Pb is concerned, whereas Al, Cd, Cu, Fe, Mn, and Ni, which were > 1, cannot be consumed safely.

## 5. Conclusions

This study showed consistent interspecific variations in the content of essential mineral elements among the studied orange-fleshed sweetpotato genotypes, as well as important intraspecific differences, which could depend on the variations in soil mineral and organic matter content, rainfall, temperature, as well as interactions between genotype and environment (GEI). Thus, we need to consider these differences when selecting genotypes for introduction of sweetpotato in a new production environment where sweetpotato has not previously been grown.

The investigated genotypes, especially Kulfo, Ininda, Gloria, and Amelia, can provide several essential minerals in amounts high enough to meet 100% RDA for all age groups ≤8 years. These features justify the increased potentials of OFSP in assuaging mineral deficiencies in young children. Further investigation of these promising sweetpotato genotypes in other parts of the arid agro-climate is needed to verify the need for biofortification; this will help to scale up the concentration of essential nutrients to meet the RDA for age groups ≥8 years, including pregnant and breastfeeding ladies.

The mean content of potentially toxic elements in tuberous roots and their daily intake values were within the recommended permissible levels; likewise, no health risk was associated with the consumption of these genotypes for As, Cr, and Pb, but health risks emerged for Al, Cd, Cu, Fe, Mn, Ni, and Zn, especially for age groups ≤8 years old compared to adults ≥19 years old.

## Figures and Tables

**Table 1:** Main features of the study sites.

	Mekelle-1	Mekelle-2	Aba’ala
Latitude N	13° 28′46′	13° 28′50.6′	13° 21′19′
Longitude E	39° 29′09′	39° 29′23.9′	39° 45′17′
Altitude (m asl)	2223	2223	1441
Annual rainfall (mm)	406	523	394
Min annual temperature (°C)	13.1	12.2	18.6
Max annual temperature (°C)	25.3	24.4	34.0
Soil texture	Silty clay	Silty clay	Silty-loamy
Sand (%)	18	18	28
Silt (%)	49	49	54
Clay (%)	33	33	18
pH	7.0	7.0	6.9
EC (ds/m)	0.23	0.23	0.11
CEC (cmol/kg)	41.3	41.3	38.0
OC (%)	0.48	0.52	1.90
Total N (%)	0.09	0.10	0.14
Available K (ppm)	134	134	121
Available P (ppm)	26.6	26.6	38.7
Fe (ppm)	11.0	11.0	15.1
Zn (ppm)	5.4	5.4	6.5
Ca (ppm)	29.4	29.4	25.4

**Table 2:** Average weight, height, and body mass for different age groups used to determine the RDA/RAI for different essential nutrients is taken from U.S. National Academy of Sciences [33].

Gender	Age	Body Mass Index, kg m^−2^	Height, cm (in)	Weight kg (lb)
Male and female	6–12 months	—	72 (28)	9 (20)
	4–8 years	15.8	118 (46)	22 (48)
Male	19–30 years	22.6	176 (69)	70 (153)
Female	19–30 years	22.6	163 (64)	60 (131)

**Table 3:** Content of essential mineral elements in sweetpotato genotypes (mean ± standard error; mg 250 g^−1^ dw) in the studied environments.

Environment	Genotype	Ca	Cu	Cr	Fe	K	Mg	Mn	Na	Ni	P	Zn
Aba’ala	Amelia	486 ± 2	2 ± 0	0.18 ± 0.01	10 ± 1	3555 ± 16	204 ± 4	3 ± 0	174 ± 4	3.9 ± 0.1	580 ± 6	4 ± 1
Aba’ala	Gloria	273 ± 10	2 ± 0	0.25 ± 0.01	9 ± 3	3675 ± 14	99 ± 2	2 ± 0	76 ± 3	3.7 ± 0.1	407 ± 3	4 ± 0
Aba’ala	Ininda	179 ± 7	2 ± 0	0.18 ± 0.01	9 ± 2	4272 ± 18	100 ± 2	1 ± 0	72 ± 3	2.9 ± 0.1	507 ± 5	4 ± 0
Aba’ala	Kulfo	372 ± 8	2 ± 0	0.19 ± 0.01	10 ± 3	1568 ± 11	274 ± 7	2 ± 0	431 ± 6	3.7 ± 0.1	480 ± 4	4 ± 1
Aba’ala	Melinda	508 ± 1	2 ± 0	0.15 ± 0.01	10 ± 3	2099 ± 13	274 ± 6	2 ± 0	391 ± 6	2.2 ± 0.1	539 ± 5	4 ± 1
Mekelle-1	Amelia	340 ± 3	2 ± 0	0.15 ± 0.01	10 ± 2	1034 ± 5	225 ± 4	1 ± 0	543 ± 4	2.5 ± 0.1	348 ± 8	3 ± 1
Mekelle-1	Gloria	278 ± 11	1 ± 0	0.19 ± 0.01	10 ± 2	1333 ± 9	147 ± 5	1 ± 0	392 ± 4	1.6 ± 0.1	292 ± 5	3 ± 0
Mekelle-1	Ininda	230 ± 8	2 ± 0	0.17 ± 0.01	10 ± 1	1604 ± 4	158 ± 5	2 ± 0	297 ± 5	2.2 ± 0.1	344 ± 6	3 ± 0
Mekelle-1	Kulfo	242 ± 9	1 ± 0	0.18 ± 0.01	10 ± 1	1157 ± 10	167 ± 3	1 ± 0	675 ± 4	1.8 ± 0.1	342 ± 5	3 ± 1
Mekelle-1	Melinda	689 ± 8	2 ± 0	0.13 ± 0.01	11 ± 3	2458 ± 9	248 ± 4	2 ± 0	329 ± 7	0.8 ± 0.1	568 ± 7	4 ± 1
Mekelle-2	Amelia	379 ± 8	2 ± 0	0.13 ± 0.01	11 ± 1	1524 ± 6	262 ± 4	2 ± 0	395 ± 6	1.7 ± 0.1	452 ± 6	4 ± 1
Mekelle-2	Gloria	174 ± 12	1 ± 0	0.27 ± 0.01	13 ± 1	968 ± 6	142 ± 5	1 ± 0	380 ± 5	1.0 ± 0.1	234 ± 4	4 ± 1
Mekelle-2	Ininda	175 ± 10	2 ± 0	0.23 ± 0.01	11 ± 1	1502 ± 5	127 ± 5	1 ± 0	210 ± 5	1.5 ± 0.1	356 ± 5	3 ± 0
Mekelle-2	Kulfo	219 ± 10	2 ± 0	0.24 ± 0.01	11 ± 2	1126 ± 9	167 ± 3	1 ± 0	607 ± 5	1.2 ± 0.1	337 ± 4	4 ± 1
Mekelle-2	Melinda	271 ± 2	2 ± 0	0.15 ± 0.01	11 ± 1	1288 ± 7	226 ± 4	2 ± 0	358 ± 4	2.5 ± 0.1	389 ± 5	3 ± 1

**Table 4:** Two-way ANOVA of mineral elements (* = *p* < 0.05).

Source of Variation	Ca	Cu	Cr	Fe	K	Mg	Mn	Na	Ni	P	Zn
Genotype	2,127,795 *	6.19 *	0.01 *	24 *	25,695,899 *	461,967 *	29 *	2,825,824 *	0.95	749,608 *	34
Environment	1,090,146 *	10.07 *	0.01	202 *	217,030,847 *	1937	24 *	3,083,098 *	13.32 *	1,525,083 *	81
Interaction	625,759 *	2.17 *	0.00	111 *	45,047,780 *	150,500 *	38 *	386,427 *	1.58	47,337	20
Residual	40,652	0.3	0.00	6	1,256,471	2744	0.5	1296	1.64	33,431	27

**Table 5:** Intake of essential mineral elements in OFSP genotypes with a daily dose of 250 g of d.w. of orange flesh sweetpotato per person (70 kg body weight) in the studied environments.

Essential Elements	RDA/RAI (mg d^−1^)	Age Group	Genotypes				
			Amelia	Gloria	Ininda	Kulfo	Melinda
	270	6–12 months	149	90	72	103	181
	800	4–8 years	50	30	24	35	61
Ca (mg/250 g)	1000	19 years >	40	24	19	28	49
	1300	Pregnant ladies 30–50 years	31	19	15	21	61
	1300	Lactation 19–50 years	31	19	15	21	61
	0.5	6–12 months	360	300	380	320	380
	0.4	4–8 years	450	375	475	400	475
Cu (mg/250 g)	0.7	19 years >	257	214	271	229	271
	0.77	Pregnant ladies 30–50 years	234	195	247	208	247
	1.3	Lactation 19–50 years	138	115	146	123	146
	5.5	6–12 months	3.0	4.0	3.5	3.6	2.9
	15	4–8 years	1.1	1.5	1.3	1.3	1.1
Cr (mg/250 g)	25	19 years >	0.7	0.9	0.8	0.8	0.6
	30	Pregnant ladies 30–50 years	0.5	0.7	0.6	0.7	0.5
	45	Lactation 19–50 years	0.4	0.5	0.4	0.4	0.4
	40	6–12 months	26	27	26	25	27
	40	4–8 years	26	27	26	25	27
Fe (mg/250 g)	45	19 years >	23	24	23	22	24
	45	Pregnant ladies 30–50 years	23	24	23	22	24
	45	Lactation 19–50 years	23	24	23	22	24
	500	6–12 months	387	388	381	400	389
	400	4–8 years	484	485	476	499	486
K (mg/250 g)	700	19 years >	277	277	272	285	278
	770	Pregnant ladies 30–50 years	252	252	247	259	252
	1300	Lactation 19–50 years	149	149	146	154	150
	75	0–12 months)	307	172	171	270	333
	130	4–8 years	177	99	98	156	192
Mg (mg/250 g)	320	19 years >	72	40	40	63	78
	400	Pregnant ladies 30–50 years	58	32	31	51	62
	360	Lactation 19–50 years	64	36	35	56	69
	0.6	6–12 months	350	233	200	200	317
	3	4–8 years	70	47	40	40	63
Mn (mg/250 g)	11	19 years >	19	13	11	11	17
	11	Pregnant ladies 30–50 years	19	13	11	11	17
	11	Lactation 19–50 years	19	13	11	11	17
	500	6–12 months	74	43	43	108	72
	400	4–8 years	92	73	54	135	90
Na (mg/250 g)	700	19 years >	53	42	31	77	51
	770	Pregnant ladies 30–50 years	48	38	28	70	47
	1300	Lactation 19–50 years	28	22	21	42	28
Ni (mg/250 g)	ND	6–12 months	ND	ND	ND	ND	ND
	0.3	4–8 years	133	200	200	167	200
	0.6	19 years >	67	100	100	84	100
	1	Pregnant ladies 30–50 years	40	60	60	50	60
	1	Lactation 19–50 years	40	60	60	50	60
	275	6–12 months	167	113	146	140	181
	500	4–8 years	92	62	80	77	100
P (mg/250 g)	1250	19 years >	37	25	32	31	40
	1250	Pregnant ladies 30–50 years	37	25	32	31	40
	1250	Lactation 19–50 years	37	25	32	31	40
	3	6–12 months	117	113	117	113	113
	5	4–8 years	70	68	70	68	68
Zn (mg/250 g)	11	19 years >	32	31	32	31	31
	13	Pregnant ladies 30–50 years	27	26	27	26	26
	14	Lactation 19–50 years	25	24	25	24	24

ND = not determined by US National Academy of Sciences [33] and Trumbo et al. [32].

**Table 6:** Content (mg kg^−1^ dw) of potentially toxic elements in sweetpotato genotypes in the studied environments. Different letters indicate statistical differences between the genotypes.

Genotype	Al	As	Cd	Cr	Cu	Fe	Mn	Ni	Pb	Zn
Amelia	18.5 ab	0.025 b	0.14 a	0.16 b	7.4 a	41.0 ab	8.5 a	2.7 a	0.40 a	15.1 a
Gloria	21.8 a	0.033 a	0.22 a	0.24 a	5.9 b	43.2 a	5.4 c	2.1 a	0.48 a	11.4 a
Ininda	19.0 ab	0.031 a	0.18 a	0.19 ab	7.7 a	40.8 b	4.7 cd	2.2 a	0.44 a	16.3 a
Kulfo	16.6 b	0.033 a	0.22 a	0.20 ab	6.4 b	40.0 b	4.7 d	2.2 a	0.61 a	12.5 a
Melinda	16.7 b	0.033 a	0.12 a	0.15 b	7.7 a	43.8 a	7.7 b	1.8 a	0.63 a	13.7 a
CV	3.2	8.1	8.1	4.1	1.6	1.3	1.8	4.8	8.4	9.1
Environment										
Aba’ala	17.8 a	0.026 b	0.19 a	0.19 a	8.0 a	38.7 b	7.7 a	3.3 a	0.62 a	15.3 a
Mekelle-1	18.8 a	0.037 a	0.15 a	0.16 a	7.4 b	40.7 b	5.3 b	1.8 b	0.46 a	11.6 a
Mekelle-2	18.9 a	0.029 ab	0.18 a	0.21 a	7.3 b	45.8 a	5.6 b	1.6 b	0.46 a	13.5 a

**Table 7:** Two-way ANOVA of potentially toxic elements (* = *p* < 0.05).

Source of Variations	Al	As	Cd	Cr	Cu	Fe	Mn	Ni	Pb	Zn
Genotype	41.03 *	0.00	0.02	0.01 *	6.19 *	24.38 *	29.09 *	0.95	0.10	34.24
Environment	5.45	0.00 *	0.01	0.01	10.07 *	202.97 *	24.10 *	13.32 *	0.14	81.14
Interaction	22.52	0.00	0.00	0.00	1.33 *	59.21 *	20.09 *	1.46	0.12	24.72
Residual	9.47	0.00	0.01	0.00	0.33	6.56	0.25	1.64	0.12	28.22

**Table 8:** Daily Intake of PTEs (DIPTEs, mg) through the intake of 250 g d^−1^ (dw) of the studied sweetpotato genotypes.

	Al	As	Cd	Cr	Cu	Fe	Mn	Ni	Pb	Zn
Amelia	5.6	0.008	0.04	0.04	1.8	12.4	2.6	0.82	0.01	4.6
Gloria	6.6	0.010	0.07	0.04	1.6	13.1	1.6	0.63	0.01	3.5
Ininda	5.8	0.009	0.05	0.05	1.9	12.4	1.4	0.66	0.01	5.0
Kulfo	5.0	0.010	0.07	0.06	1.5	12.2	1.4	0.67	0.02	3.8
Melinda	5.1	0.009	0.04	0.05	1.9	13.3	2.4	0.55	0.02	4.2

**Table 9:** Health Risk index (*HRI*) of PTEs from oral exposure for each studied sweetpotato genotypes.

Genotypes	Al	As	Cd	Cr	Cu	Fe	Mn	Ni	Pb	Zn
Amelia	5.60	0.11	40.00	0.03	45.00	17.71	185.71	41.00	0.40	15.33
Gloria	6.60	0.14	70.00	0.03	40.00	18.71	114.29	31.50	0.40	11.67
Ininda	5.80	0.13	50.00	0.03	47.50	17.71	100.00	33.00	0.40	16.67
Kulfo	5.00	0.14	70.00	0.04	37.50	17.43	100.00	33.50	0.80	12.67
Melinda	5.10	0.13	40.00	0.03	47.50	19.00	171.43	27.50	0.80	14.00

## Data Availability

The raw data presented in this study are available on request from the corresponding author.
